# A multimodal deep learning approach for the prediction of cognitive decline and its effectiveness in clinical trials for Alzheimer’s disease

**DOI:** 10.1038/s41398-024-02819-w

**Published:** 2024-02-21

**Authors:** Caihua Wang, Hisateru Tachimori, Hiroyuki Yamaguchi, Atsushi Sekiguchi, Yuanzhong Li, Yuichi Yamashita, for Alzheimer’s Disease Neuroimaging Initiative

**Affiliations:** 1 Bio Science & Engineering Laboratories, FUJIFILM Corporation, Ashigarakami-gun, Kanagawa Japan; 2 Department of Information Medicine, National Institute of Neuroscience, National Center of Neurology and Psychiatry, Tokyo, Japan; 3 Endowed Course for Health System Innovation, Keio University School of Medicine, Tokyo, Japan; 4 Department of Psychiatry, Yokohama City University School of Medicine, Yokohama, Japan; 5 Department of Behavioral Medicine, National Institute of Mental Health, National Center of Neurology and Psychiatry, Tokyo, Japan

**Keywords:** Neuroscience, Predictive markers

## Abstract

Alzheimer’s disease is one of the most important health-care challenges in the world. For decades, numerous efforts have been made to develop therapeutics for Alzheimer’s disease, but most clinical trials have failed to show significant treatment effects on slowing or halting cognitive decline. Among several challenges in such trials, one recently noticed but unsolved is biased allocation of fast and slow cognitive decliners to treatment and placebo groups during randomization caused by the large individual variation in the speed of cognitive decline. This allocation bias directly results in either over- or underestimation of the treatment effect from the outcome of the trial. In this study, we propose a stratified randomization method using the degree of cognitive decline predicted by an artificial intelligence model as a stratification index to suppress the allocation bias in randomization and evaluate its effectiveness by simulation using ADNI data set.

## Introduction

Alzheimer’s disease (AD) is an irreversible neurodegenerative disease that causes brain cells to degenerate, and its symptoms, such as memory impairment, greatly impact the activities of daily living of affected patients. As of 2022, there were an estimated 6.1 million patients with AD, and this number is estimated to grow to 13.8 million by 2060 in the USA alone [1]. On the other hand, AD has few available treatments, and there has been a high rate of failure in AD drug development programs [2]. Those trials have often failed to show efficacy in slowing or halting cognitive decline [3, 4]. Participants with mild cognitive impairment (MCI) who are in the prodromal stage of AD are generally involved in clinical trials. For such trials, one of the most often used end points is the change in the Clinical Dementia Rating-Sum of Boxes (CDR-SB) score, which has been shown to be able to stage the severity of both AD and MCI accurately [5, 6].

There are many difficulties in clinical trials for AD [7]. For trials using cognitive decline as an end point, two challenges are associated with the end point itself. One generally known challenge is that due to the large individual variance in the speed of cognitive decline, which has been suggested in previous studies [8–10], the recruited participants usually contain a considerable proportion of slow decliners or non-decliners. For those participants having small or no cognitive decline, the effects of treatment on suppression of cognitive decline, which can be expected to be on only a portion of the decline, will be even smaller or null and weaken the total observed effect of treatment on all participants in the trial. Several previous studies have aimed to select fast decliners for clinical trials, and various methods, including machine learning, have been proposed to predict disease progression [11–17]. Because selecting fast decliners for a trial may involve the uncertainty of whether a drug is actually more effective for fast rather than slow decliners, a more appropriate way of using such methods for participant selection would be to exclude only non-decliners from trials.

Another impact of the large individual variance in the speed of cognitive decline in clinical trials of AD is that it may cause biased allocation of fast and slow decliners to treatment and placebo groups in the randomization procedure, which could result in the over-/underestimation of treatment effects in the trial outcomes. Allocation biases in cognitive decline between treatment and placebo groups appear directly in the outcome of a trial as an over-/underestimated effect of the treatment. Although other biases exist in allocated groups of participants, such as adverse events and individual responses to treatment, may also result in over-/underestimation of the trial outcome, allocation bias in cognitive decline remains the dominant challenge because of the large variation in the speed of cognitive decline compared with the outcomes of previous trials. Therefore, suppressing the allocation bias in cognitive decline could be expected to suppress the over-/underestimation of trial outcomes. The influence of allocation biases in cognitive decline on clinical trials for AD in which cognitive decline is used as the end point was recently investigated [18]; however, this problem has yet to be solved.

**Fig. 1:**
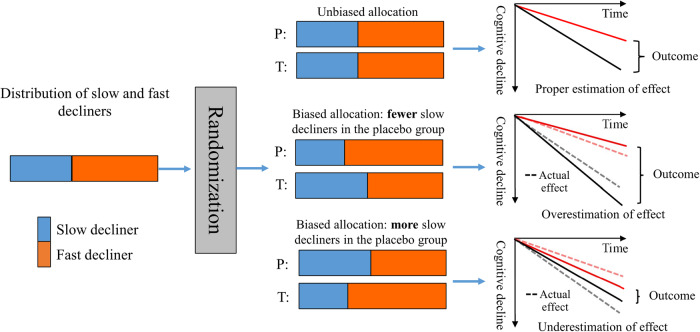
Three cases of biases occurred in allocating participants to the treatment and placebo groups (T and P in the figure, respectively) in clinical trials. Solid lines in the right part of the figure represent the cognitive decline in the treatment and placebo groups observed during the trial period, and dashed lines represent the cognitive decline in the two groups when there were no allocation bias and no treatment applied. When the participants were unbiasedly allocated to the treatment and placebo groups, the treatment effect could be properly evaluated with the outcome of the trial. Otherwise, when more fast cognitive decliners were allocated to the placebo group, the treatment effect was over-evaluated, and vice versa in the opposite case. The over-/underestimated effects were equal to the allocation bias between the treatment and placebo groups.

As shown in Fig. 1, when participants are allocated to treatment and placebo groups unbiasedly, that is, when the slow and fast cognitive decliners allocated for treatment and placebo groups have the same ratio, the treatment effect can be estimated properly based on the trial outcome. When fast cognitive decliners are allocated more to the placebo group and less to the treatment group, the cognitive scores of the participants in the placebo group decline more than that in the treatment group. In this case, the effect of treatment will be overestimated based on the trial outcome. An overestimation of the treatment effect in an earlier phase of the trial will lead to an underestimation of the necessary sample size for the next phase, which may lead to failure in the next phase of the trial. On the other hand, when slow cognitive decliners are allocated more to the placebo group and less to the treatment group, the effect of treatment will be underestimated based on the trial outcome. An underestimation of the treatment effects of the trial can result in the wrong decision regarding dropping a promising drug that could benefit patients. Therefore, an unbiased allocation of fast and slow cognitive decliners into treatment and placebo groups is an important task for clinical trials on AD using cognitive decline as the end point.

Generally, using risk factors of AD related to cognitive decline, such as biomarkers of amyloid beta 42 (Aβ42), phosphorylated Tau (pTau), and apolipoprotein E (ApoE) ε4, as a stratification index in randomization can reduce the allocation biases in cognitive decline. ApoE ε4 has already been used for stratification randomization in some recent trials [19–21]. As individual risk factors have limited predictive power for cognitive decline, a recent survey on the prediction of disease progression in AD suggested the possibility of utilizing artificial intelligence (AI) technology to reduce allocation biases [22].

**Fig. 2:**
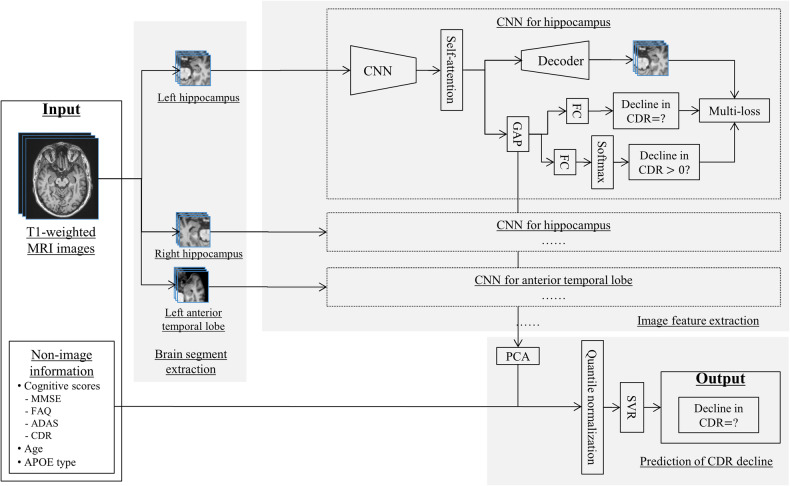
A hybrid multimodal machine learning framework for predicting CDR-SB changes. Multiple DNN models were trained to extract image features from several subregions of the brain related to cognitive decline, such as the hippocampus and anterior temporal lobe. A multitask loss including image recovery, decliner and non-decliner classification, and CDR-SB changes regression was used to train the DNN models to extract the image features robustly. The extracted image features and non-image information were then used in linear support vector machine regression (SVR) to predict CDR-SB changes.

**Fig. 3:**
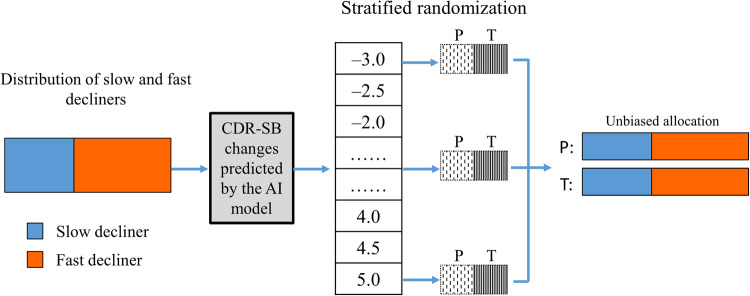
Scheme of stratified randomization using predictions of CDR-SB changes outputted by the AI model. According to the predicted CDR-SB changes, the participants were first stratified into subgroups (or strata). The participants in each subgroup (or stratum) were then randomly allocated into treatment and placebo groups (T and P in the figure, respectively) in equal numbers. The participants allocated to the treatment groups in all stratified subgroups was collected to the treatment group of the trial, and the others to the placebo group.

The purpose of this study was to propose an AI-based approach for randomization procedure of clinical trials for AD and to evaluate its impact of allocation bias reduction on trial efficiency. First, we developed a hybrid multimodal deep learning model, as shown in Fig. 2, to predict CDR-SB changes during the trial period using both image information including T1-weighted images and non-image information including demographic information, cognitive test scores and biomarkers available at baseline. This predictive model was extended from a previously proposed hybrid multimodal deep learning model [15] that achieved high performance for the prediction of disease progression from MCI to AD. Next, as shown in Fig. 3, we proposed an AI-based randomization method in which the prediction of cognitive decline outputted by the AI model was used as the stratification index in stratified randomization [23, 24]. Finally, we evaluated the impact of the AI-based randomization method on trial efficiency by simulating different randomization methods using the North American Alzheimer’s Disease Neuroimaging Initiative [25] (NA-ADNI) data set, and quantitatively comparing their effectiveness in allocation bias reduction and power on treatment effect detection. Compared to conventional non-stratified randomization, the proposed AI-based randomization could reduce the allocation bias by approximately 22%, resulting in a reduction in sample size of nearly 37%.

## Results

### Accuracy of prediction of CDR-SB changes using the AI model

**Fig. 4:**
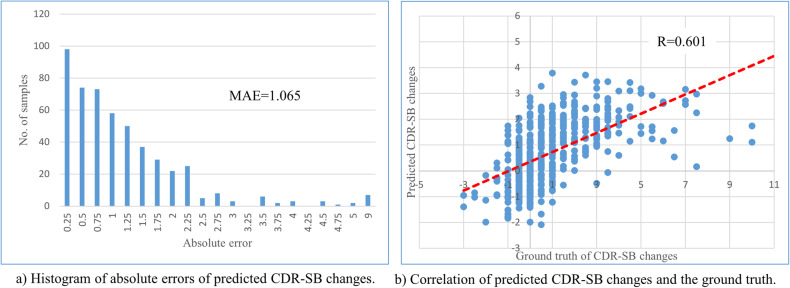
Prediction results of the samples used for randomization simulation. **a** Histogram of absolute errors of predicted CDR-SB changes by the AI model; (**b**) correlation of predicted CDR-SB changes and the ground truth.

We extracted 1194 samples of 506 participants from the NA-ADNI database (http://adni.loni.usc.edu/ADNI) using inclusion criteria similar to those used in recent trials (for details, see the Methods section). The prediction model was trained, validated, and tested using tenfold cross-validation and test setting with all the samples. Predictions of 506 samples of each participant at baseline were obtained from test sets in the tenfold cross-validation and test setting for use in the simulations. The prediction accuracies of the samples used in the simulations are shown in Fig. 4 and Table 1. The mean value and standard deviation (SD) of actual CDR-SB changes (ground truth [GT]) of all 506 samples were 0.978 and 1.899, respectively. The distributions (histogram) of the absolute error (AE) of CDR-SB changes predicted by the AI model are shown in Fig. 4a, in which the horizontal axis shows AEs with an interval of 0.25 and the vertical axis shows the number of samples whose prediction accuracy by the AI model fell into each interval. The correlation between CDR-SB changes as predicted by the AI model and the GT is shown in Fig. 4b. The mean absolute error (MAE) of the AI predictions was 1.065 and the correlation coefficient for the AI predictions and actual values was 0.601. Table 1 also shows the same information for the inner and outer samples, that is, the samples in or out of the 2σ interval of the distribution of the actual CDR-SB changes (GT) for all samples, where σ is the SD of the distribution of GT. The prediction error was larger for samples with large actual CDR-SB changes (GT), especially the predicted CDR-SB changes for the samples having large positive actual CDR-SB changes (*n* = 23), which were almost irrelevant compared with the actual values.

**Table 1:** Accuracy of the model for the samples used for randomization simulation.

Samples	SN	MAE	Correlation
All	506	1.074	0.580
Inner	481	0.892	0.620
Outer	25	4.567	–0.036

Inner/outer samples within/outside a 2*δ* interval of the distribution of the actual CDR-SB changes (ground truth [GT]) for all samples.

### Simulation results for the reduction in allocation biases

**Fig. 5:**
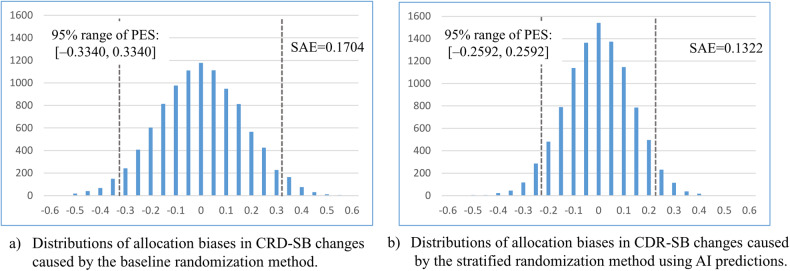
Distributions of allocation biases of CDR-SB changes. Distributions of allocation biases of CDR-SB changes in the treatment and placebo groups caused by the non-stratified randomization method (**a**) and stratified randomization method predictions of CDR-SB changes outputted by AI mode for stratification (**b**). Each distribution was obtained by simulating the corresponding randomization method 10,000 times. Dashed gray lines show the 95% range of the distributions, which corresponds to the 95% range of effect sizes possibly observed when the treatment effect was null. The effect size possibly observed when the treatment effect was null is called the possible effect size (PES) in this study.

Allocations were first simulated for a typical phase II trial with sample size of 500 using both non-stratified and stratified randomization in which the AI prediction of CDR-SB changes was used as the stratification index. Figure 5 shows the distributions of the allocation biases obtained by the two randomization methods. The SD of the distribution was used to evaluate the size of the allocation biases obtained by each randomization method (referred to in the present study as the standard allocation error [SAE]). The SAEs of the above non-stratified and stratified randomization methods were 0.1704 and 0.1322, respectively. The 95% ranges of the distributions of the allocation biases obtained by the two methods were [–0.3340, 0.3340] and [–0.2592, 0.2592] (dashed gray lines in the figure), respectively. The 95% range of the distribution of the allocation biases was equivalent to the 95% range of the effect size which could be observed when the treatment effect in the trial was null. Below, the effect size possibly observed with a null-effect treatment is referred to as the possible effect size (PES). A narrow 95% range of the PES implies a smaller priori effect size to be considered in a trial, which largely influences the design of the trial and the reliability of the evaluation of its outcome.

**Fig. 6:**
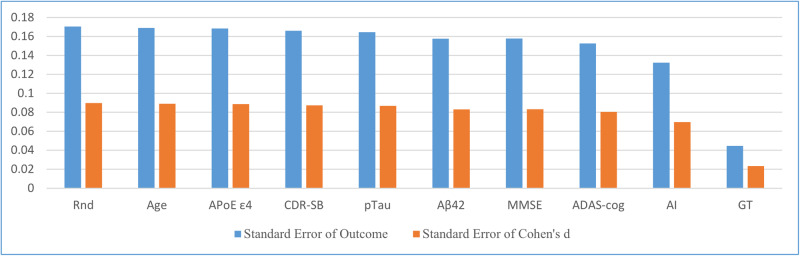
Standard allocation error (SAE) in CDR-SB changes and Cohen’s d of each randomization method. Rnd: non-stratified randomization, and age, CDR-SB, ApoE ε4, pTau, Aβ42, MMSE, and ADAS-cog: stratified randomization using each index for stratification. AI and GT: stratified randomization using predictions of the AI model and actual CDR-SB changes (ground truth [GT]) for stratification, respectively. The SAE in CDR-SB changes and that in Cohen’s d are approximately in constant proportionality for all randomization methods.

For a comprehensive comparison, stratified randomization using demographic information (age), biomarkers (ApoE ε4, Aβ42, and pTau), and cognitive scores (CDR-SB, the Mini–Mental State Examination [MMSE], and the Alzheimer’s Disease Assessment Scale–Cognitive [ADAS-cog]) as stratification indices was also simulated. Stratified randomization using actual CDR-SB changes (GT) was also simulated to provide a reference value for the allocation biases. For each randomization method, a distribution of allocation biases was obtained from the simulation, and its SD was calculated as the SAE of each method. The results of the comparison of SAEs across all randomization methods are shown in Fig. 6. The SAEs in Cohen’s d (that is, the SD of the distribution of allocation biases in Cohen’s d) of the allocation biases of all randomization methods are also shown in the figure. The SAEs in the CDR-SB changes and Cohen’s d were proportional (see Supplementary Table E1); therefore, they can be considered equivalent. As shown in the figure, the stratified randomization method using AI prediction of CDR-SB changes as a stratification index could reduce the SAE much more than the other indices. Compared with non-stratified randomization, simulations using stratified randomization with AI prediction could reduce the SAE by 22.4%, whereas using ADAS-cog, the best among the other indices, could reduce the SAE by only 10.5% (see Supplementary Table E1). On the other hand, using the stratified randomization method with actual CDR-SB changes (GT) as a stratification index could reduce the SAE by 73.9%, which strongly encourages the improvements of AI prediction model.

### Simulation results for improvements in efficiency of trial

**Fig. 7:**
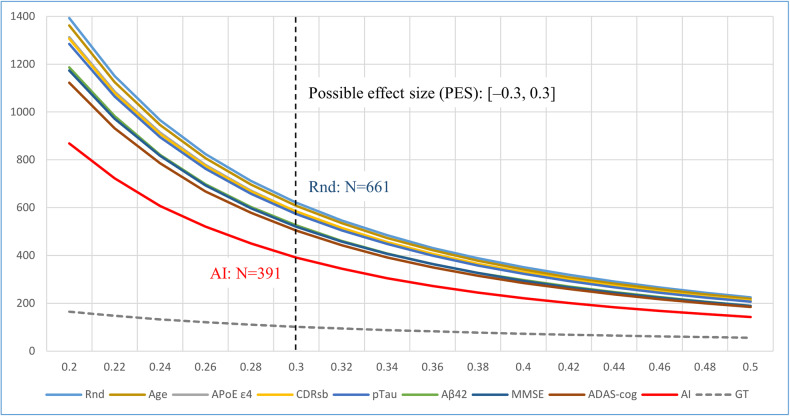
Minimum sample sizes needed for different randomization methods to obtain the same given 95% range of possible effect size (PES) in CDR-SB changes. Horizontal axis: the upper bound of the 95% range of PES. Rnd: simple randomization without stratification. Age, CDR-SB, ApoE ε4, pTau, Aβ42, MMSE, and ADAS-cog: stratified randomization using each index. AI and GT: stratified randomization using predictions by the AI model and actual values (ground truth; GT) of CDR-SB changes, respectively. For example, the minimum sample sizes for non-stratified and stratified randomization using AI predictions to obtain the same 95% range of the PES of [–0.3, 0.3] were 661 and 391, respectively.

If the 95% range of the PES is the priori effect size to be considered in a trial, then the sample sizes needed for different randomization methods to control the priori effect size in a given range are different. Figure 7 shows the minimum sample size necessary for each randomization method to obtain the same 95% range of the PES, that is, the a priori effect size to be considered in the trial. The horizontal axis in the figure shows the 95% range of the PES with its upper bound, and the vertical axis shows the sample size needed to obtain the 95% range of the PES. In the figure, we can see that for a given 95% range of the PES bounded to [–0.3, 0.3], the non-stratified randomization method needed at least 661 samples to ensure the 95% range of the PES to be within the given range, whereas the stratified randomization using predictions outputted by the AI model as a stratification index could reduce the sample size to 391, which was a 37% reduction from that using non-stratified randomization. For an additional comparison, using age as a stratification index in randomization could reduce the sample size by 2.1%, using CDR-SB, ApoE ε4, and pTau as stratification indices could reduce the sample size by 6.0–7.7%, and using Aβ42, MMSE, and ADAS-cog scores could reduce the sample size by 15.3–18.8%. When actual CDR-SB changes (GT) were used for stratification, the sample size could even be reduced by 83.6% (for more details, see Supplementary Table E2).

### Simulation results for over-/underestimation suppression in clinical trials

**Fig. 8:**
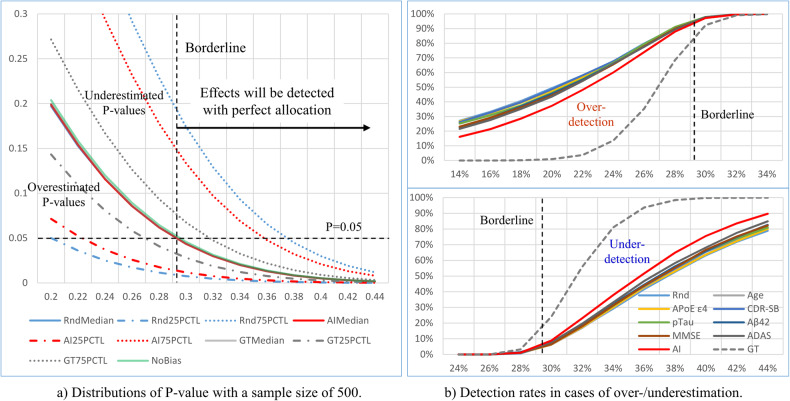
Distributions of *P* values and over-/under-detection of actual treatment effects. **a** Distributions of *P* values (shown with median, 25th and 75th percentiles) obtained by non-stratified randomization (Rnd), stratified randomizations using AI predictions (AI) and the ground truth (GT) of CDR-SB changes. The horizontal axis shows the treatment effects, defined as suppressing CDR-SB changes in the treatment group by x%, and the vertical axis shows *P* values. *P* values calculated with perfect randomization without bias (NoBias) are also shown. Medians of the *P* values of three randomization methods overlapped with that obtained with perfect randomization. The borderline of treatment effects corresponding to the threshold *P* value (*P* = 0.05) existed where the *P* value obtained by perfect randomization was equal to the threshold. Under-detection of treatment effects occurred in the 1st quadrant formed by the threshold of *P* = 0.05 and the borderline. On the other hand, over-detections occurred in the 3rd quadrant. **b** Detection rates of treatment effects applied to the treatment group, separately evaluated for two cases of whether the mean value of the GT of CDR-SB changes of the placebo was larger (above) or smaller (below) than that of the treatment group after allocation and before treatment effects were applied.

A critical problem of allocation biases is that they result in over-/underestimation of the treatment effect from trial outcomes, especially when the sample size is relatively small. In actual clinical trials, treatment effects are usually evaluated based on the *P* values of *t* tests carried out on the outcomes of placebo and treatment groups, and allocation bias influences the *P* value. Figure 8a shows the distributions of *P* values (vertical axis) of *t* tests on outcomes of trials with a sample size of 500 for several treatment effects using different randomization methods. The distributions are shown with their median and 25th and 75th percentiles. Effects were defined as suppressing the actual CDR-SB changes (GT) in the treatment group by x%, where x ranged from 10 to 50 with an interval of 2 (horizontal axis). In the figure, we can see that the medians of the *P* values obtained by each randomization method completely overlapped with those obtained by perfect allocation in which the distributions of CDR-SB changes of two groups were the same as that of the whole population; therefore, the *P* values located above/below the medians can be considered the result of over-/underestimation. The ranges from the 25th to 75th percentiles of the *P* value distributions show that the distributions of *P* values obtained by AI-based randomization were narrower than those obtained by non-stratified randomization, which implies that using AI prediction in randomization can suppress both the over-/underestimation of treatment effects compared with non-stratified randomization.

In clinical trials, treatment effects are often detected by a *P* value threshold, which was set to 0.05 in the present study. As shown in Fig. 8a, with perfect allocation, all effects larger than the borderline corresponding to the threshold are detected, whereas all those smaller than the borderline are not. With other randomization methods, due to allocation biases, over-/under-detection happens in effects smaller or larger than the borderline, respectively. Figure 8b shows the detection rates of different treatment effects applied to the treatment group using different randomization methods, separately evaluated based on two cases: whether the mean actual CDR-SB change (GT) of the placebo group were larger (above) or smaller (below) than those of the treatment group after allocation and before effect application. With randomization using AI predictions, the detection rates were improved and suppressed when the actual treatment effects were relatively large and small, respectively. For example, when the actual treatment effect was 38%, using AI-based randomization maximally improved the treatment effect detection rate, from 52.6% when non-stratified randomization was used, to 64.9%, and further to 98.5%, by using the GT of CDR-SB changes. On the other hand, when the actual treatment effect was 20%, a detection rate of 49.4% was achieved by non-stratified randomization, which was suppressed to 37.3% by stratified randomization using AI prediction and further to 1% by using the GT of CDR-SB changes. While the benefit of improved detection rates for large actual treatment effects is self-explanatory, that of suppressing detection rates when actual treatment effects are relatively small is only revealed in a multiple-phase trial; this is described further in the Discussion section and Supplementary Materials.

## Discussion

In this study, we developed a hybrid multimodal deep learning model to predict CDR-SB changes, which are frequently used as a primary end point in clinical trials of AD, by extending a previously proposed model that achieved high performance in predicting disease progression from MCI to AD. We further proposed an AI-based randomization method by using the prediction of CDR-SB changes outputted by the model as the stratification index in a stratified randomization scheme. We showed the effectiveness of the proposed randomization method in allocation bias reduction by simulation using the NA-ADNI data set. The crucial role of allocation bias in clinical trials has recently been noticed and investigated, and researchers have started to recognize that AI technology could provide a new approach to solving this problem. However, to our best knowledge, neither how to embed AI technology into allocation procedures to reduce allocation bias nor whether it can be effective in clinical trials has been investigated yet. Our study is the first to use prediction of cognitive decline by an AI model as the stratification index in stratified randomization, and to report the effectiveness of using such a randomization method in clinical trials of AD.

We also investigated the effectiveness of the AI-based randomization when participant selection was simultaneously applied. Previous studies have suggested that selecting fast progressors of AD for a clinical trial might improve trial outcomes [16, 17]. On the other hand, fast progressors generally have relatively large individual variation in cognitive decline, as shown in Supplementary Table E3, where slow and fast progressors were classified by the disease progression risk factors of age, ApoE ε4, and cerebrospinal fluid (CSF) pTau. The large variation in cognitive decline usually results in a large allocation bias, and this was already shown in a previous study [18] in which allocation biases in low- and high-risk groups were investigated by simulation. Here, we reproduced the same simulation as that in the previous study but added a simulation of randomization using AI prediction for the fast progressor group. To maintain a fair comparison, all simulations were carried out with a fixed sample size of 500 (oversampling was applied for subgroups with fewer than 500 samples). The simulation results are shown in Supplementary Fig. E1. With non-stratified randomization, the results indicated that the SAEs (as well as the 95% range of the PES, that is, the effect size possibly observed when the treatment effect is null) obtained from fast decliners classified by the risk factors of age, ApoE ε4, and pTau (e–g) were larger than those obtained from all participants containing both slow and fast decliners (a). In addition, a reversal occurred for slow decliners (b–d). Using the stratified randomization with AI prediction as a stratification index, the distributions of allocation biases obtained from the fast decliners revealed that the 95% ranges of the PESs obtained from the fast decliners classified by the risk factors of age, ApoE ε4, and pTau were 0.271, 0.299, and 0.294, respectively (h–j). All were smaller than the 95% range of the PES obtained for all participants containing both slow and fast decliners (0.334) when non-stratified randomization was used.

As shown in the Results section (Fig. 8b), when the treatment effect was smaller than the borderline where the *P* value obtained by perfect randomization was equal to the threshold of *P* = 0.05, the detection rates of the treatment effects were suppressed by stratified randomization using AI prediction for stratification. On the other hand, when the treatment effect was larger than the borderline, the detection rates were improved. As shown in Supplementary Fig. E2, when the sample size became larger, the borderline became lower. This suggests that when the sample size is relatively small, using AI-based randomization may suppress over-detection of a wide range of treatment effects (Fig. 8b above), resulting in suppressing the underestimation of the sample size for the next phase of the trial, but when the sample size was relatively large, as shown in Supplementary Fig. E3a, the detection rate was improved for a wide range of treatment effects by using AI-based randomization, resulting in an improved success rate for a large-scale trial. On the other hand, the over detection rate was suppressed by AI-based randomization only in a narrow range of small treatment effects (Supplementary Fig. E3b).

When a trial with multiple phases was considered, the early phase, such as a proof-of-concept phase, was often conducted with a relatively small sample size, followed by a large-scale phase for which the sample size was estimated from the outcome of the early phase. Supplementary Fig. E4 shows the simulation results for a multiple-phase trial, where the early phase was conducted with a sample size of 500, and the late phase with a sample size estimated from the observed effective size (OES) or outcome of the early phase. Using AI prediction as a stratification index in randomization improved the total success rate when the actual effect was larger than 18%. For example, when the actual effect was 30%, using AI prediction in randomization was improved from 52.1% to 60.8% compared with non-stratified randomization. When the actual treatment was small, that is, smaller than 18%, non-stratified randomization performed slightly better than AI-based randomization, although neither method had much chance of success. Details of the simulation can be found in the Methods section.

In the present study, we found that using AD risk factors such as age, ApoE ε4, biomarkers (amyloid β42 and pTau), and cognitive scores (CDR-SB, MMSE, and ADAS-cog) as stratification indices in stratified randomization could also reduce the allocation bias in CDR-SB changes to a certain degree. One obvious explanation for this is that the risk factors related to the progression of AD are also related to declines in CDR-SB scores; therefore, using these risk factors as stratification indices in stratified randomization could reduce the allocation bias in CDR-SB changes. Supplementary Fig. 5E shows the relationship between the SAEs obtained by randomization methods and the correlation coefficients between the stratification indices used in randomization and the actual CDR-SB changes (GT). The results show that the SAEs and correlation coefficients were reversely proportional to each other (Supplementary Fig. E5a), and highly correlated to each other with a correlation coefficient of −0.969 (Supplementary Fig. E5b), which implies that stratification indices with higher correlations for cognitive decline generally result in smaller allocation biases.

This study did have some limitations. First, all of our simulations and results were based on the NA-ADNI data set, in which samples were primarily collected from Europid populations. Using only the NA-ADNI data set may restrict the generalizability of our results to the global population or other cohorts. Second, although the NA-ADNI study was designed to reflect a potential clinical trial population, and we selected samples using recruitment criteria similar to those used in the most recent trials for simulations, our approach should be further evaluated with actual trial data. Third, a lot of trials, especially phase II trials, used end points other than CDR-SB changes, such as biomarkers, as the primary end point, so a different prediction model should be developed and validated for other types of end points. In addition, in our simulations, we considered only trials with two arms and a single stratification index. The effectiveness of using AI prediction in more complicated trial designs, such as those with multiple arms, randomization with stratification on multiple indices, and/or advanced adaptive randomization, should be further investigated.

For the prediction model, there remains room for improvement. Cognitive decline used as primary end points in AD trials is typically evaluated using neuropsychological tests, which usually contain noise, so a regression model for such noisy targets remains an open challenge. In the present study, we introduced multitask loss, that is, we combined regression loss with classification and image recovery losses, to make robust predictions of noisy targets. However, large prediction errors still exist for the outlying samples, as shown in Table 1. Therefore, more sophisticated robust losses should be considered. To overcome another challenge in our study, which is a lack of enough training data, we constructed a hybrid framework that proved to be effective for predicting the progression of MCI to AD when the available data were limited [15]. However, end-to-end models, including those using other state-of-the-art architectures such as vision transformer as backbones, should be continuously investigated because projects like the NA-ADNI are ongoing and data sets will continue becoming larger.

## Methods

### Data sets used in this study

The data used in this study were obtained from the ADNI-1, ADNI-GO, and ADNI-2 data sets in the NA-ADNI database (http://adni.loni.usc.edu/ADNI). The NA-ADNI is a cohort study launched in 2003 as a public–private partnership, led by principal investigator Michael W. Weiner M.D., and carried out across 55 research centers in the USA and Canada. The clinical coordination center of the NA-ADNI established a network of clinical sites and developed a plan for the recruitment and retention of subjects, and furthermore, prepared the final clinical protocol and informed consent, which were distributed to each site for local institutional review board approval [25]. All subjects were willing and able to undergo all testing procedures, including neuroimaging and follow-up, and to provide written informed consent. Over 2000 participants with normal cognition and patients with MCI or AD were recruited for the present study. The first cohort, referred to as ADNI-1, consisted of 800 individuals: 200 cognitively normal (CN) individuals, 400 with late MCI, and 200 with mild dementia. ADNI-GO, the second cohort, included about 200 additional individuals with early MCI. In ADNI-2, more participants at different stages of AD were recruited to monitor the progression of AD. ADNI-3, which is presently enrolling additional CN individuals and patients with MCI and dementia, was not included in our study, because no diagnosis information is currently available. The ethical approval for this study was given by both FUJIFILM Corporation and National Center of Neurology and Psychiatry.

The hybrid model was trained, validated, and tested using samples from longitudinal data in the NA-ADNI data set selected based on the following criteria: (1) a clinical diagnosis of MCI or AD with an MMSE score ≥24; (2) amyloid beta-positive in CSF or on positron emission tomography imaging; (3) a global CDR scale score of 0.5; and (4) had the information needed for model training and an available 2-year follow-up CDR-SB score. A total of 1194 samples from 506 participants were used for model training, validation, and testing, and 506 samples at the baseline visit of each participant were used for the randomization simulation. Characteristics at baseline of the participants used in simulation were shown in Supplementary Table E4. Predictions of CDR-SB changes used for the simulation were obtained from the test sets in a participant-based tenfold cross-validation test setting.

### Image normalization and brain segment extraction

The three-dimensional (3D) T1-weighted magnetic resonance images (MRI) used in our study were first transformed into Montreal Neurological Institute (MNI) [26] space by aligning them with atlas images (standard images) created in MNI space. The template images of the MNI152 NLIN 2009a atlas [27] were used. A coarse-to-fine approach containing landmark- and image registration-based linear alignment was used to align the T1-weighted MRI images, which were obtained under protocols used in the NA-ADNI and J-ADNI studies, robustly and accurately to the template images of the atlas, which were created in MNI space. In this method, six landmarks in the brain with distinct local anatomic structures were first detected from the T1-weighted MRI images using a region-based convolutional neural network [28]. Then, an initial linear alignment between the T1-weighted MRI and atlas images was obtained from the correspondences of the detected landmarks in the T1-weighted MRI images and that predefined for the atlas images. The initial alignment was further refined using an image registration technique that utilizes mutual information as an image similarity metric [29–31].

As T1-weighted MRI images acquired in different sites with different equipment may have different biases in terms of intensity distribution, image intensity normalization is an important pre-procession for many image analysis tasks. In this study, we adopted a similar example-based intensity normalization approach that uses patches of the acquired T1-weighted MRI images and corresponding patches of an atlas template image that contain tissue contrasts desired for normalized patches in T1-weighted MRI images [32]. Instead of normalizing the global intensity distribution of whole brain images, this method normalizes the intensity distributions of local patches of T1-weighted MRI images to those of atlas images. To assure that the local intensity distributions used for the intensity normalization were calculated on same anatomic structure in the T1-weighted MRI and atlas images, the linear alignment between the T1-weighted MRI and atlas images was further refined using nonlinear (i.e., B-Spline) alignment, and local intensity distributions were calculated based on nonlinearly aligned patches. More details about the shape and intensity normalization of T1-weighted images can be found in a previous study [15].

After the intensity normalization was carried out, a skull-stripping process was performed on the shape-normalized images to extract the brain regions. To achieve this, we trained a V-net [33] with four layers using the same data set as that used for the landmark detection.

From the normalized (in shape and intensity) and skull-stripped image, brain segments of the hippocampus (left and right) and anterior temporal lobes (left and right) were extracted. The locations of these segments were manually identified in the atlas template image, and their sizes were fixed to 64 × 64 × 64 voxels, which was large enough to contain each segment of interest with a necessary margin in the normalized image. For each sample, four brain segments, which contained hippocampi and anterior temporal lobes, both left and right, at the same locations specified in the atlas template images, were extracted from its normalized image, respectively.

### Training deep neural networks (DNNs) for image feature extraction

Two DNN models of the same structure were trained to extract image features from brain segments of hippocampi (left and right) and anterior temporal lobes (left and right), respectively. The DNN models adopted a densely connected 3D convolutional network (DenseNet3D), extended form DenseNet-121 [34], as a backbone. To improve the stability of the DNN models on limited training samples, we added a self-attention (SA) [35] layer and an auto-encoder (AE) [36, 37] to the backbone, because SA can highlight important positions in a feature map and help extract the global relationship, and an AE task, which is considered more robust than classification and regression with a small number of samples, can be expected to improve the robustness of the entire architecture of the model. More details of DNN models can be found a previous study [15].

A mixed loss function of the multitask framework, defined as follows, was used to optimize the training parameters of the DNN models:

1 $${\rm{Loss}}=(1-{\rm{\beta }})(\alpha {L}_{{regression}}+\left(1-\alpha \right){L}_{{class}})+\beta {L}_{{AE}}$$

where *L*_*regression*_ is the MAE for the continuous prediction of CDR-SB changes, *L*_*class*_ represents the classification error function defined by the cross-entropy loss for the binary prediction of whether CDR-SB changes were greater than 0, *L*_*AE*_ denotes the image recovery error function defined by smooth L1 loss, and *α* and β are weight parameters, both of which being set to 0.8 empirically. As the regression task is generally sensitive to label noise, we combined it with a binary classification task that has a similar purpose, but is more robust to label noise.

The two DNN models were trained independently on the brain segments of hippocampus and the anterior temporal lobe. Because the left and right segments of the brain are basically symmetrical, images of the left hippocampus were flipped horizontally and then used with images of the right hippocampus to train the hippocampus DNN model; this process was also applied to the anterior temporal lobe segment. The output of the global average pooling of the trained network was extracted as image features and used with other nonimage features for final prediction, as described below.

### Linear support vector regression (SVR)

Because the number of training samples in our study was limited, we adopted a hybrid framework in which linear support vector regression (SVR) was used as the final predictor of CDR-SB changes instead of end-to-end deep learning. After a 128-dimension image feature was extracted from each brain segment by the DNN models, principal component analysis was used to reduce the dimension to 1 for each segment. Then, nonimage information, including cognitive scores (MMSE, CDR-SB, and ADAS-cog), age, and biomarkers (ApoE ε4, Aβ42, and pTau), was combined with the reduced image features of the four segments as the SVR input. Here, we transformed ApoE type to a value representing AD risk based on the following rules [38]: for ε2/ε3, value = 0.6; for ε3/ε3, value = 1.0; for ε2/ε4 and ε3/ε4, value = 3.2; and for ε4/ε4, value = 11.6. Because the distributions of these multimodal inputs differ greatly, we used quartile normalization to normalize the combined features before applying SVR. For validation and testing, the same quartile normalization used for training was applied to the combined features. The SVR predictor outputted a continuous value of CDR-SB changes for each participant using image features and nonimage information at baseline.

### Allocation simulation

In most trials of AD, multi-arms for different doses or combinations of therapies are adopted in the design [3, 39–43] to improve the efficiency of the trial. In this study, because we concentrated on the effectiveness of introducing prediction AI into the randomization procedure, for simplicity, we considered only the trial of two arms containing placebo and treatment with single therapy and simulated a non-stratified randomization method and nine stratified randomization methods using age, ApoE ε4, amyloid β42, pTau, CDR-SB, MMSE, ADAS-cog, CDR-SB changes as predicted by an AI model, and actual CDR-SB changes (GT) as stratification indices. Block randomization [24, 44] was used to ensure that the numbers of samples allocated to placebo and treatment groups were balanced. For simplicity, in our simulation, we used blocks containing two permutation blocks of [P, T] and [T, P], where P and T stand for placebo and treatment, respectively.

Allocation was simulated for each randomization method with a sample size of 500. In each simulation, randomization tables consisting of randomly selected permutation blocks were first generated. Then, 500 participants were randomly extracted from the total population consisting of 506 participants, and allocated to placebo and treatment groups according to the randomization tables generated above. For the non-stratified randomization method, a randomization table containing a random sequence of 250 permutation blocks was generated and used. With the randomization table consisting of two permutation blocks, 500 randomly extracted samples were allocated to two groups at an exact ratio of 1:1.

For each stratified randomization method, a randomization table with a maximum length of 250 permutation blocks was first generated for each stratification subgroup (or stratum), which was defined by predetermined ranges of the stratification index. Randomly extracted samples were first separated into subgroups (or strata), and then participants in each subgroup were allocated to placebo and treatment groups according to the randomization table generated for the subgroup. Participants in the placebo groups of all subgroups were selected to obtain the placebo group for the trial, and the same was done for the treatment group. As the number of participants in a subgroup may be odd, the ratio of the number of participants in the two groups was kept to approximately 1:1. The minima, maxima, and interval used to define the subgroups of stratification indices are shown in Supplementary Table E5. Because the predictions of CDR-SB changes outputted by the AI model were continuous values, and the actual CDR-SB changes were discrete values, the minima for the subgroup definition was shifted by 0.25, which was half the interval of actual CDR-SB changes.

### Calculation of the SAE

After allocation, we obtained two groups of participants, each of which contained approximately 250 participants. Then, we calculated the mean values of actual CDR-SB changes during the follow-up period for both groups. When a perfect allocation was obtained, the mean values of actual CDR-SB changes of the two groups were the same. However, in the case of an existing allocation bias, the mean values of actual CDR-SB changes (GT) of the two groups differed from each other. The difference in the mean values of GT of the two groups was regarded as the allocation bias caused by the randomization method used. As the participants in the allocation simulation were randomly extracted, the allocation bias will change based on the type of simulation. To examine statistically the allocation bias of each randomization method, we repeated the simulation 10,000 times (as was done in a previous study [18]) for each randomization method to obtain a distribution of allocation biases. The SD of the distribution of allocation biases obtained after 10,000 simulations was calculated (referred to as the SAE in this study). The 95% range of PESs, which were the effect sizes possibly observed with a null-effect treatment, obtained by each randomization method was then calculated as [−1.96σ, 1.96σ], where σ was the SAE obtained by the randomization method.

### Calculation of sample sizes for non-null-effect conviction

For a given randomization method, the 95% range of the PES depends on the sample size, that is, a large sample size results in a small 95% range. To calculate the sample size of a given 95% range of the PES, a simulation of sample sizes up to 3000 was carried out for each randomization method. In each simulation, allocation biases in two allocated groups were recorded for each sample size from 50 to 3000 (a sufficiently large sample size compared with most trials) when a randomly extracted participant was allocated. The simulation for each method was repeated 10,000 times to obtain a distribution of allocation biases for each sample size from 50 to 3000. When the number of samples exceeded the number of participants in the total population, oversampling was used. From the distribution of allocation biases of each sample size, an SAE was calculated. For a given 95% range of the PES, the minimum sample size was identified from the SAEs calculated above as follows:

2 $$\hat{s}=\mathop{\min }\limits_{s}\left\{s{\rm{|}}1.96\times {SAE}\left(s\right) < r\right\}$$

where *r* is the 95% range of the OES and *s* is the sample size.

### Simulations of trials with actual treatment effects

Trials for different actual treatment effects were simulated for a typical phase II trial with a sample size of 500 using the different randomization methods described in the allocation simulation subsection. Supplementary Fig. E6 shows a flowchart of the simulation. Randomly extracted participants were first allocated to placebo and treatment groups using different randomization methods. Then, an assumed actual treatment effect was applied to the distribution of actual CDR-SB changes in the treatment group. The actual treatment effect was assumed to suppress the total CDR-SB changes in the treatment group by x%. Here, x ranged from 10 to 50 with a step of 2. A *t* test was carried out on two distributions of CDR-SB changes for the placebo and treatment groups, and the *P* value of the *t* test was calculated. Without loss of generality, a two-tailed *t* test was used. The above procedure was repeated 10,000 times to obtain a distribution of *P* values for each randomization method and actual treatment effect. Additionally, we also calculated *P* values for the different actual treatment effects using a perfect allocation, that is, the means and variances of CDR-SB changes for both treatment and placebo groups, without applying a treatment effect to the treatment group, were the same as that of the total population. Whereas the *P* values obtained by the perfect randomization method were uniquely determined for a given actual treatment effect, those obtained by other randomization methods were distributed around the median, which were identical to the *P* values obtained by perfect randomization. The distributions of *P* values are represented by their medians and 25th and 75th percentiles. Simulations were carried out for all the assumed actual treatment effects described above using the randomization methods described in the Results section.

The detection rates of the actual treatment effects were calculated for two cases: (1) the mean value of CDR-SB changes in the placebo group was smaller than that in the treatment group without applying a treatment effect, and (2) the reverse. Whereas the former was much more likely to cause underestimation of the OES (or outcome) of the trial, the latter was much more likely to cause overestimation.

### Simulation of trials with multiple phases

A multiple-phase trial, consisting of a proof-of-concept phase and an effect-confirming phase, was simulated for different actual treatment effects using different randomization methods. The proof-of-concept phase with a sample size of 500 was first simulated as described in the previous subsection. As the purpose of the trial was to show the priority of treatment to placebo, we used a one-tail *t* test in the multiple-phase trial simulation. The *P* value threshold to judge whether the treatment effect was significant was set to 0.1 for the proof-of-concept phase and to 0.05 for the effect-confirming phase. The effect-confirming phase was conducted only when the *P* value obtained in the proof-of-concept phase was less than the threshold of 0.1. The sample size of the effect-confirming phase was estimated based on the OES (or outcome) of the proof-of-concept phase for the next phase to achieve a *P* value of 0.01 to include an extra number of samples to that needed for threshold of 0.05. The effect-confirming phase was then conducted using the estimated sample size, and was judged as successful if the *P* value of the *t* test for the outcome was less than 0.05. The above simulation was repeated 10000 times for each randomization method to calculate a ratio of successful cases in which both the proof-of-concept and effect-confirming phases succeeded.

### Supplementary information

Supplementary Material

## Data Availability

The data used for model training, validation, and testing are publicly available at the following URLs: 1. NA-ADNI data set: http://adni.loni.usc.edu/ 2. J-ADNI data set: https://humandbs.biosciencedbc.jp/en/hum0043-v1.

## Code availability

The DenseNet code, which was used as the backbone of our architecture, is available at https://github.com/liuzhuang13/DenseNet. The Faster R-CNN code used for brain landmark detection is available at https://github.com/rbgirshick/py-faster-rcnn. The Insight Toolkit ITK, on which our image registration algorithm was implemented, is available at https://itk.org.
