# Supplementary material for: A multimodal deep learning approach for the prediction of cognitive decline and its effectiveness in clinical trials for Alzheimer’s disease
Source: Transl Psychiatry. 2024 Feb 21;14:105. doi: 10.1038/s41398-024-02819-w (PMC10882004; doi:10.1038/s41398-024-02819-w)
Supplement: Supplementary file 1 — Supplementary Material [file 41398_2024_2819_MOESM1_ESM.pdf]

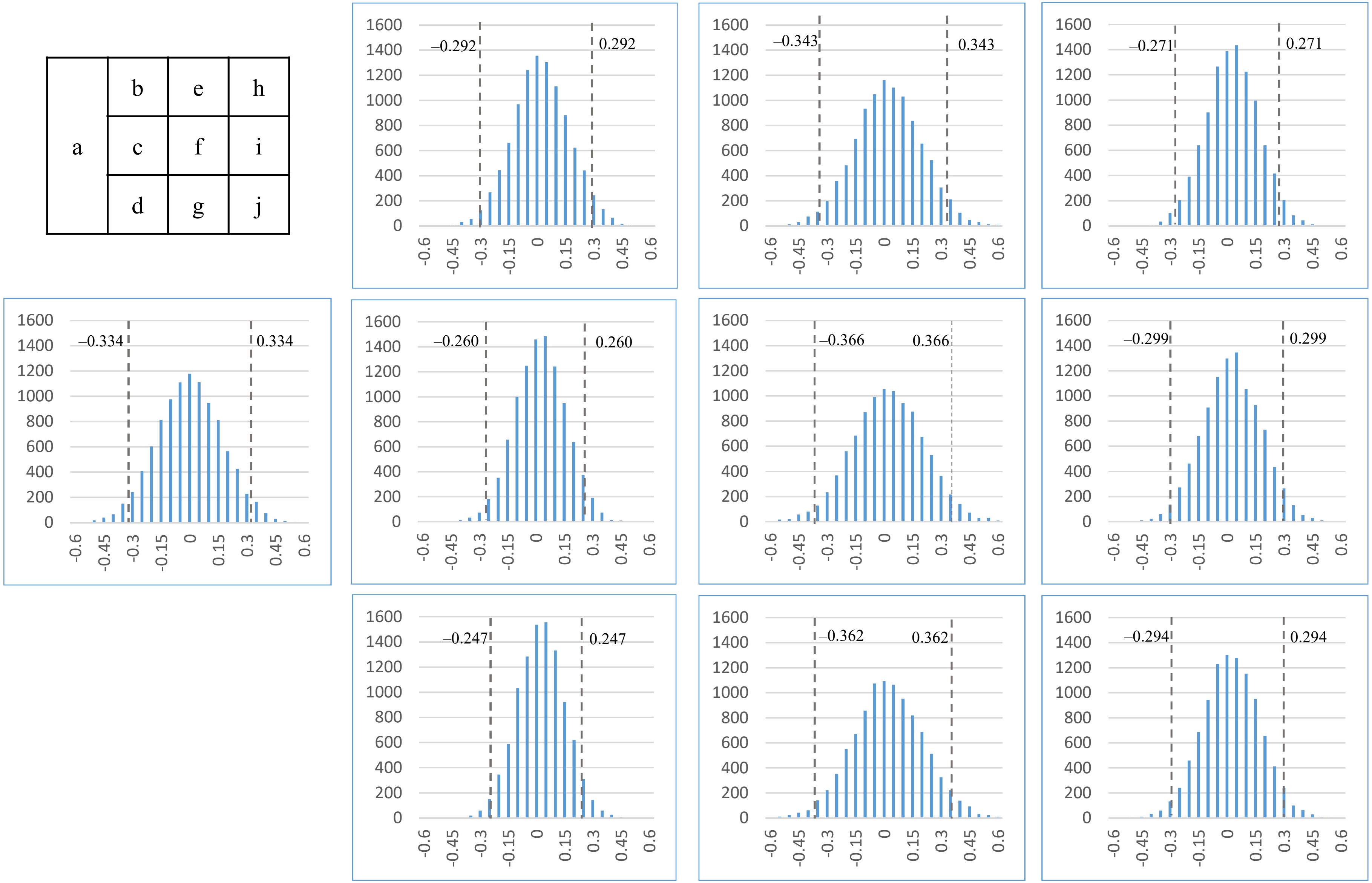


Supplementary Figure E1 Distributions of allocation biases and their 95% ranges of the PES in CDR-SB changes. a) Allocation biases obtained from the participants, including both slow and fast decliners. b–d) Allocation biases obtained from slow decliners classified by risk factors of age, ApoE ε4, and pTau, respectively. e–g) Allocation biases obtained from fast decliners. All distributions of allocation biases from a) to g) were obtained with non-stratified randomization. h–j) Allocation biases obtained from fast decliners, with stratified randomization using AI predictions as the stratification index.

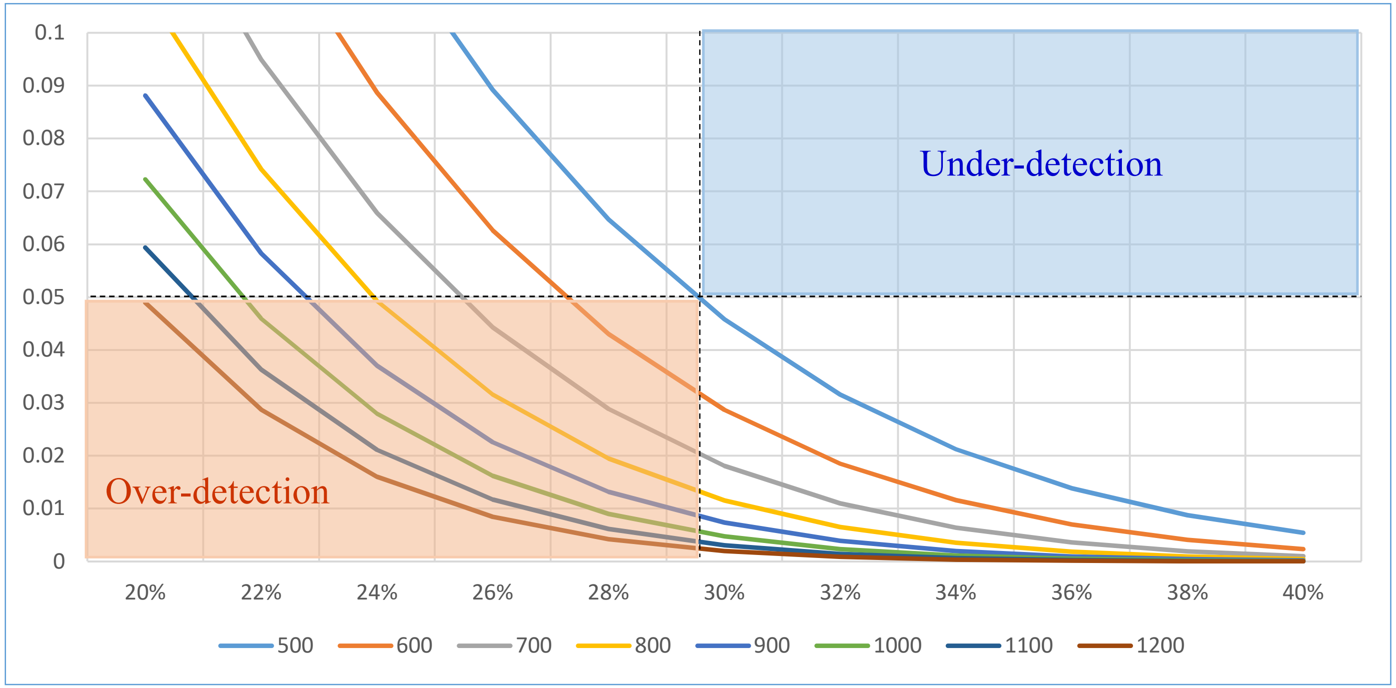


Supplementary Figure E2 P-values of *t*-tests for CDR-SB changes in the placebo and treatment groups with different sample sizes from 500 to 1200 using perfect allocation. The horizontal axis shows treatment effects, and the vertical axis shows P-values. When the sample size became large, the borderline where the P-value was equal to the threshold of P=0.05 became small. When the sample size was 1200, the borderline was smaller than 20%.

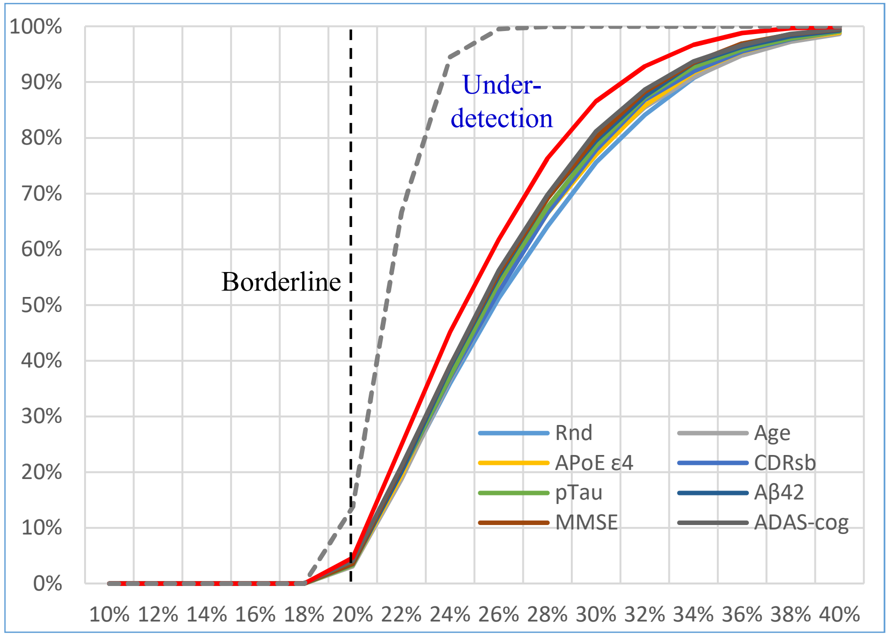


a) Detection rates in the case of underestimation of OES.

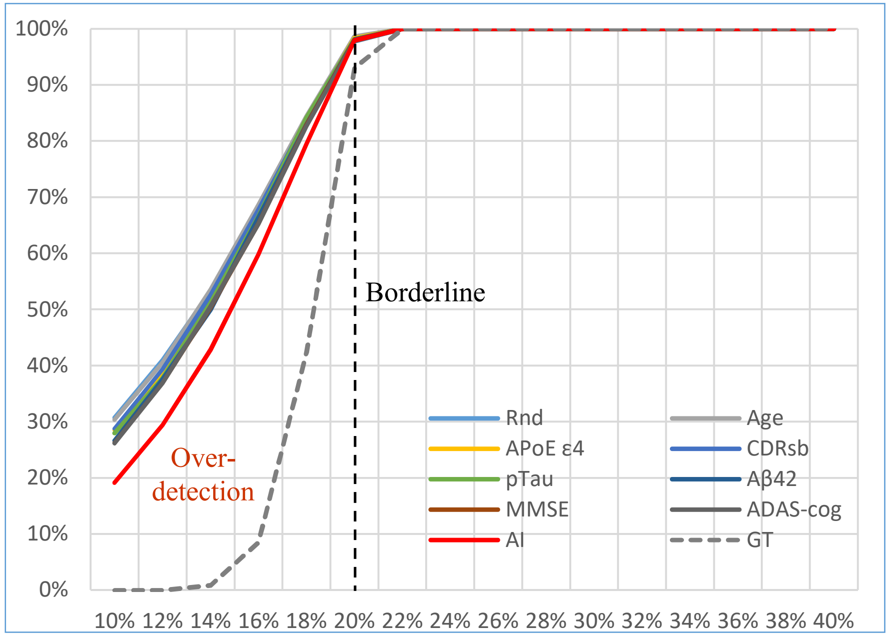


b) Detection rates in the case of overestimation of OES.

Supplementary Figure E3 Detection rates of actual treatment effects for the case when the mean value of CDR-SB changes in the placebo group was smaller than that in the treatment group without applying treatment effects (left), and the reverse (right), when the sample size was 1200. a) Detection rates in the case when the mean value of CDR-SB changes in the placebo group was smaller than that in the treatment group without applying treatment effects. b) Detection rates in the reversal case of a). The horizontal axis shows actual treatment effects, and the vertical axis shows the detection rate (%).

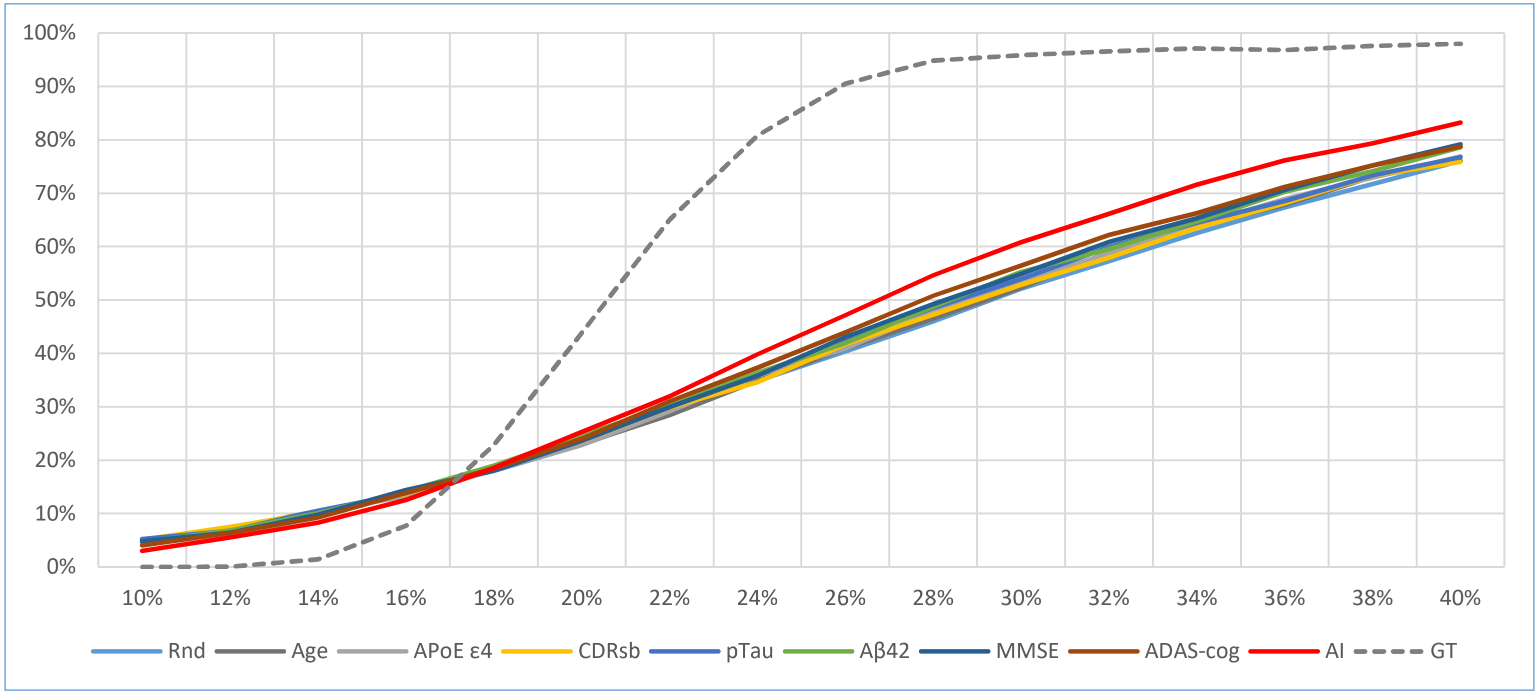


Supplementary Figure E4 Success rates of a multiple-phase trial, when the early phase was conducted with a fixed sample size of 500 and the late phase was conducted based on the success of the early phase, with a sample size estimated using the OES (or outcome) of the early phase. Using AI predictions of CDR-SB changes as a stratification index in stratified randomization improved the success rate of a multiple-phase trial when the actual treatment effect was relatively large. Non-stratified randomization performed better as a chance finder when the actual treatment effect was small.

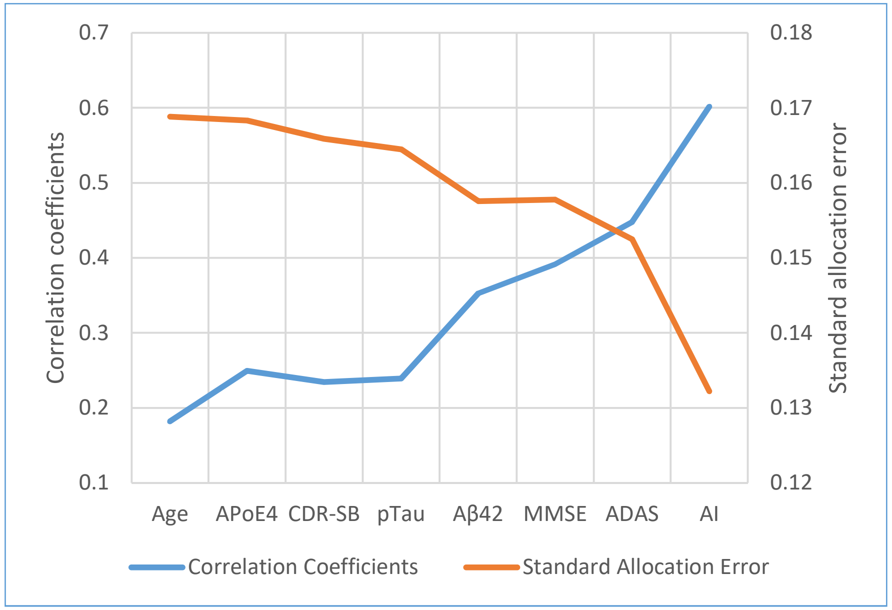


a) SDs of distributions of allocation biases.

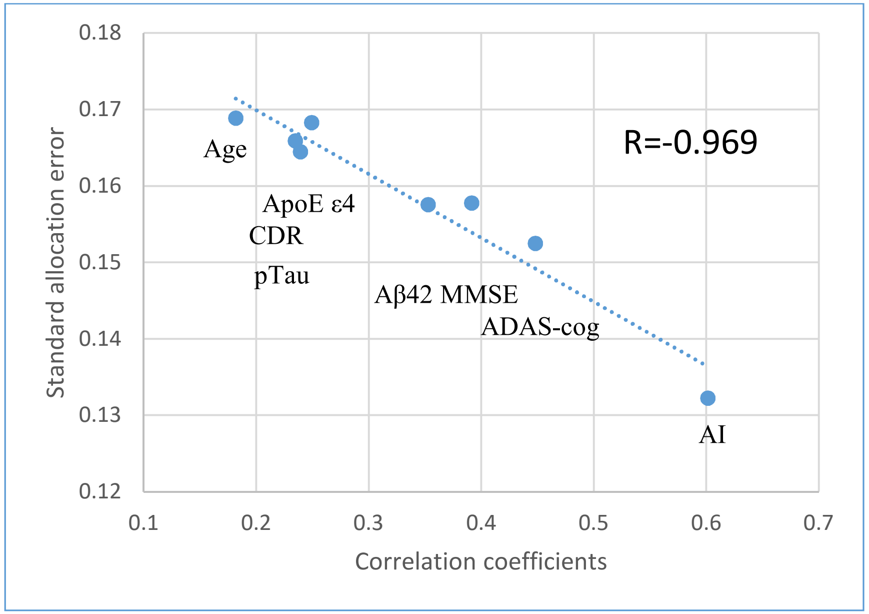


b) Linear regression of correlation coefficients and SDs of distributions.

Supplementary Figure E5 Relationship between standard allocation errors (SAEs) of stratified randomization methods and correlation coefficients of stratification indices and actual values of CDR-SB changes. a) SAEs of stratified randomization methods and correlation coefficients of stratification indices and actual values of CDR-SB changes. b) Results of linear regression of SAEs of stratified randomization methods to correlation coefficients of stratification indices and actual values of CDR-SB changes. Age, CDR-SB, ApoE ε4, pTau, Aβ42, MMSE, and ADAS-cog: stratified randomization using each index. AI: stratified randomization using predictions by the AI model. Note 1: The absolute value of the correlation coefficient of Aβ42 and the actual CDR-SB changes were used. Note 2: Samples with an abnormal pTau value > 100 (6 samples) and Aβ42 value > 300 (3 samples) were excluded from the calculation of correlation coefficients.

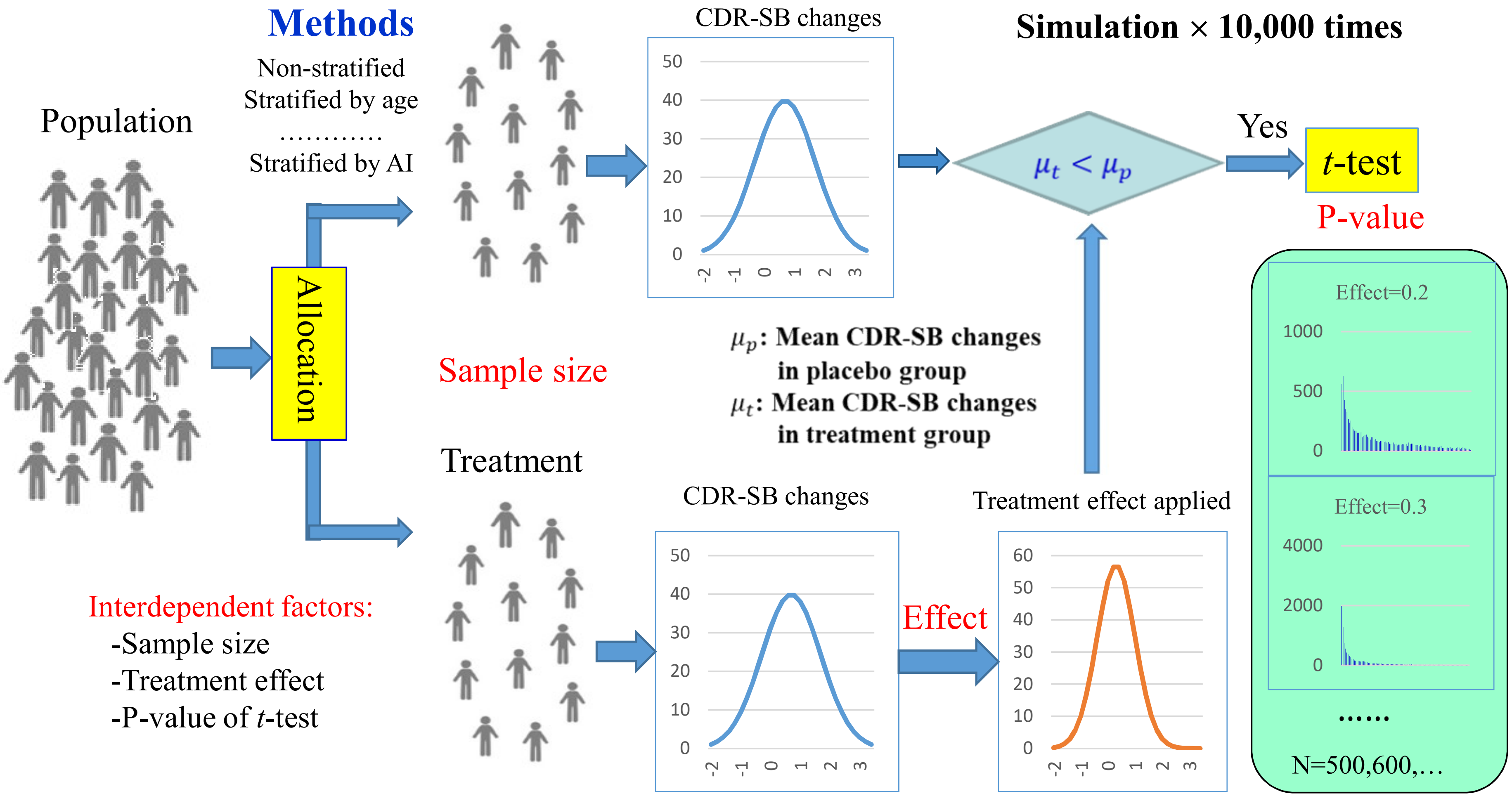


Supplementary Figure E6 Flowchart of simulations for trials with actual treatment effects. Participants with a given sample size were randomly extracted from the total population of 506 samples and then allocated to placebo and treatment groups by a given randomization method. The distribution of actual CDR-SB changes in the treatment group was calculated, and a given treatment effect was applied to the distribution to simulate the treatment. Finally, *t*-tests were carried out on the distributions of CDR-SB changes in the two groups, and a P-value was calculated. The simulation was repeated 10,000 times to obtain a distribution of P-values for each randomization method, sample size, and treatment effect.

Supplementary Table E1 Standard allocation errors (SAEs) caused by different randomization methods when the sample size was 500. The top two rows show SAEs calculated for raw CDR-SB changes and Cohen's d, respectively. The third row shows the ratio of SAEs in Cohen's d to that in raw CDR-SB changes. The last row shows reduction rates in the percentages of stratified randomization methods compared with non-stratified randomization.

| Method | Non-stratified | Age | ApoE ε4 | CDR-SB | pTau | Aβ42 | MMSE | ADAS-cog | AI | GT |
|---|---|---|---|---|---|---|---|---|---|---|
| SAE | 0.1704 | 0.1689 | 0.1683 | 0.1659 | 0.1645 | 0.1576 | 0.1578 | 0.1525 | 0.1322 | 0.0445 |
| Cohen's d | 0.0897 | 0.0889 | 0.0886 | 0.0873 | 0.0867 | 0.0830 | 0.0831 | 0.0804 | 0.0697 | 0.0233 |
| Cohen's d/SAE | 0.5264 | 0.5265 | 0.5263 | 0.5265 | 0.5269 | 0.5269 | 0.5269 | 0.5269 | 0.5269 | 0.5250 |
| Reduction rate | --- | 0.91% | 1.23% | 2.66% | 3.48% | 7.53% | 7.41% | 10.50% | 22.40% | 73.91% |

Supplementary Table E2 Minimum sample sizes needed for different randomization methods to obtain the same given 95% range of the PES. The bottom row shows reduction rates in percentages of stratified randomization methods compared with non-stratified randomization.

| | Rnd | Age | ApoE ε4 | CDR-SB | pTau | Aβ42 | MMSE | ADS-Cog | AI | GT |
|---|---|---|---|---|---|---|---|---|---|---|
| No. of samples | 621 | 608 | 583 | 584 | 573 | 526 | 520 | 504 | 391 | 102 |
| Reduction rate | --- | 2.1% | 6.1% | 6.0% | 7.7% | 15.3% | 16.3% | 18.8% | 37.0% | 83.6% |

Supplementary Table E3 Mean values and standard deviations of CDR-SB changes for slow and fast decliners classified by the risk factors of age, ApoE ε4, and pTau.

| | Total | Age | | ApoE ε4 | | pTau | |
|---|---|---|---|---|---|---|---|
| | | <70 years | ≥70 years | Non-carrier | Carrier | Negative | Positive |
| SN | 506 | 189 | 317 | 232 | 274 | 179 | 327 |
| Mean | 0.978 | 0.614 | 1.196 | 0.517 | 1.369 | 0.344 | 1.326 |
| Standard deviation | 1.899 | 1.681 | 1.988 | 1.496 | 2.107 | 1.412 | 2.038 |

Supplementary Table E4 Characteristics of the participants at baseline (N=506)

| Characteristics | Number or Mean±StDev |
|---|---|
| Age | 72.3±7.45 |
| Gender(Male/Female) | 290/216 |
| AopE ε4 (Noncarrier/Carrier) | 232/274 |
| MMSE | 27.6±1.86 |
| CDR-SB | 1.57±0.95 |
| ADAS-cog | 10.2±6.62 |
| Amyloid β42 | 136.5±48.8 |
| pTau | 40.9±21.9 |

Supplementary Table E5 Minima, maxima, and interval for stratifying each index into subgroups (strata).

| | Gender | Age | CDR-SB | ApoE ε4 | pTau | Aβ42 | MMSE | ADAS-cog | AI |
|---|---|---|---|---|---|---|---|---|---|
| Minima | 0 | 50 | 0.5 | 0 | 10 | 80 | 24 | 0 | –2.25 |
| Maxima | 1 | 100 | 5 | 2 | 100 | 300 | 30 | 45 | 4.25 |
| Interval | 1 | 10 | 0.5 | 1 | 10 | 20 | 1 | 5 | 0.5 |
